# Gelling Behavior of PAM/Phenolic Crosslinked Gel and Its Profile Control in a Low-Temperature and High-Salinity Reservoir

**DOI:** 10.3390/gels8070433

**Published:** 2022-07-11

**Authors:** Fei Ding, Caili Dai, Yongpeng Sun, Guang Zhao, Qing You, Yifei Liu

**Affiliations:** 1 School of Petroleum Engineering, China University of Petroleum (East China), No. 66 Changjiang West Road, Huangdao District, Qingdao 266580, China; b18020025@s.upc.edu.cn (F.D.); sunyongpeng@upc.edu.cn (Y.S.); zhaoguang@upc.edu.cn (G.Z.); liuyifei@upc.edu.cn (Y.L.); 2 School of Energy Resources, China University of Geosciences (Beijing), No. 29 Xueyuan Road, Haidian District, Beijing 100083, China; youqing@cugb.edu.cn

**Keywords:** low temperature, high salinity, conformance control, salt resistance, phenolic, gel

## Abstract

Gel conformance control technology is widely used in moderate and high temperature reservoirs. However, there are few studies on shallow low-temperature and high-salinity reservoirs. The difficulties are that it is difficult to crosslink at low temperatures and with poor stability at high salt concentrations. Therefore, the PHRO gel was developed, which was composed of gelatinizing agent (polyacrylamide), crosslinking agents (hexamethylenetetramine and resorcinol) and crosslinking promoting agent (oxalic acid). The PHRO could form high-strength gels in both deionized water and high-concentration salinity solutions (NaCl, KCl, CaCl_2_ and MgCl_2_). The observation of the microstructure of PHRO gel shows that a strong “stem—leaf”-shaped three-dimensional network structure is formed in deionized water, and the network structure is still intact in high-concentration salt solution. The results show that PHRO has good salt resistance properties and is suitable for conformance control of low-temperature and high-salinity reservoirs.

## 1. Introduction

Waterflooding is a common method for the displacement of crude oil. It also has the function of a formation energy supplement. It is an effective way for oilfields to maintain stable production and is widely used worldwide. Nonetheless, with the development of water injection in the middle and late stages, the formation heterogeneity is aggravated by flushing with long-term water injection, and the injected water enters the production well along the large-pore or high-permeability zone. As a result, oilfields are facing problems of increased water cuts and declining oil production. There is still a considerable amount of remaining oil irregularly distributed in the unswept area of reservoirs by waterflooding [1,2]. Therefore, enhancing oil recovery in the middle and late stages of waterflooding development has become an important research topic for oilfield development researchers.

The North Buzachi oilfield in Kazakhstan is located on the Buzachi peninsula in the northeastern Caspian Sea [3]. The reservoir depth is less than 500 m. The average porosity is 32.4%. The average permeability is 1930 mD. The reservoir temperature is 29–33 °C, and the total salinity of formation water is 3.78 × 10^4^ − 6.09 × 10^4^ mg/L. The North Buzachi oilfield is encountering a decline in the formation pressure coefficient, a high water–oil ratio and declining oil production in some wells after a long period of waterflooding. To improve waterflooding performance and ensure stable oil production, it is necessary to plug large channels or highly permeable zones to expand the swept volume of injected water.

The practice of oilfield development in the past 20 years shows that the most effective conformance control method is the application of chemical plugging agents [4]. The commonly used plugging agents are resin, foam, gel, etc., and gel is the most frequently applied among them [5,6,7,8,9,10,11]. Gel is a type of plugging agent with a three-dimensional network structure formed through a crosslinking reaction between gelation agents and crosslinking agents [12,13]. Gelation agents are water-soluble polymers, such as polyacrylamide and polyethylene imine, of which polyacrylamide and its derivatives are the most commonly used. For crosslinking agents, there are two main types [14,15,16,17]: (1) organic metal crosslinking agents, such as Cr^3+^, Zr^4+^ and Al^3+^, can be crosslinked with the carboxylic acid group in the polymer molecule; (2) organic crosslinking agents, such as formaldehyde-phenol and phenolic resin, can be crosslinked with amide groups in polymer molecules. At present, research on gel focuses on middle and deep reservoirs, whereas there are few studies on shallow reservoirs with low temperature and high salinity.

Due to the low activity of crosslinking agents at low temperatures, the crosslinking reaction of most gels is slow or does not occur. Chromium and phenolic gels are the most commonly used gels at low temperatures [18,19]. Chromium gels are composed of PAM and Cr^3+^ crosslinking agents, which have the characteristics of fast gelation and high strength at low temperatures [11,20,21,22,23]. However, they result in high water loss due to structural damage in high-salinity water over time. Then, the stability and strength of chromium gels will decline sharply, which will greatly reduce the plugging period. DiGiacomo [24] found that in the presence of calcium and magnesium ions, the dehydration of chromium gels was intensified and suggested that this was due to Ca^2+^ and Mg^2+^ ions reacting with the carboxyl group in PAM and producing carboxylates with low water solubility. Wang [25] investigated the impact of calcium chloride on the dehydration behavior and the microstructure of PAM/Cr^3+^ gel. The experimental results indicated that a high concentration of calcium chloride could promote the water loss of gels, while a low concentration of calcium chloride could inhibit the dehydration of gels. The reduction in water-holding holes in PAM/Cr^3+^ gel is the internal reason that a high concentration of calcium chloride aggravates the water loss of gels.

In this study, a phenolic crosslinking gel was prepared, referring to the temperature and salinity of formation water in the North Buzachi oilfield. The fluid consists of polyacrylamide, hexamethylenetetramine, resorcinol and oxalic acid (PHRO). The oxalic acid has dual functions of accelerating crosslinking and shielding saline ions, which can reduce the gelation time and strengthen the salt resistance of PHRO. First, the influence of different additive concentrations on gelling performance and fluid stability was studied. Secondly, the gelling performance and stability of the fluid in different concentrations of sodium chloride, potassium chloride, calcium chloride and magnesium chloride solutions were studied. Finally, the salt tolerance mechanism of the fluid was analyzed from the microscopic morphology. The results demonstrate that PHRO is suitable for conformance control in low-temperature and high-salinity conditions. This study provides basic theoretical support for the development of conformance control technology in shallow reservoirs with low temperatures and high salinity.

## 2. Results and Discussion

### 2.1. Preparation of PHRO Gel

#### 2.1.1. Process of Crosslinking

The crosslinking reactions of PHRO gel are condensation reactions between polyacrylamide (PAM), hexamethylenetetramine (HMTA) and resorcinol (RO). There are three types of crosslinking reactions [17]:

1. HMTA is hydrolyzed to produce formaldehyde, which is crosslinked with PAM;
2. Formaldehyde reacts with RO to generate polyhydroxy methyl phenol, which is crosslinked with PAM;
3. Formaldehyde reacts with RO to phenolic resin, which is crosslinked with PAM.

**Figure 1:**
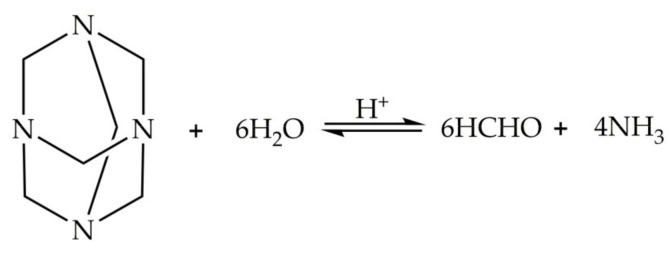
Hydrolytic process of HMTA.

The hydrolysis of HMTA to formaldehyde is the key step in the formation of the gel, and the reaction process is illustrated in Figure 1. The hydrolysis reaction is a reversible reaction, and the reaction rate is very slow at low temperatures. The addition of OA makes the solution acidic, which shortens the gelation time of PHRO.

#### 2.1.2. Macroscopic and Microscopic Morphology of Gel

**Figure 2:**
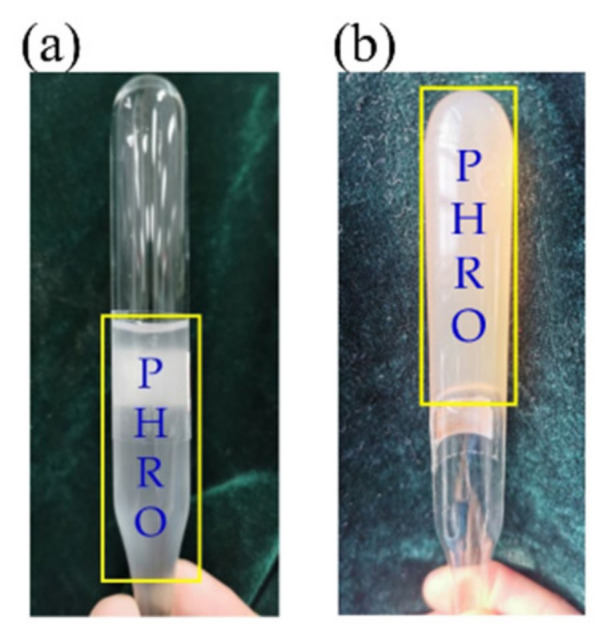
Picture of PHRO before and after crosslinking. (**a**) Before crosslinking; (**b**) after crosslinking (the formula is 0.4 wt% PAM + 0.5 wt% HMTA + 0.05 wt% RO + 0.3 wt% OA).

The appearance of the PHRO gel in deionized water is shown in Figure 2. The gel formed in ionized water is dark orange, and its strength reaches the “G” level.

**Figure 3:**
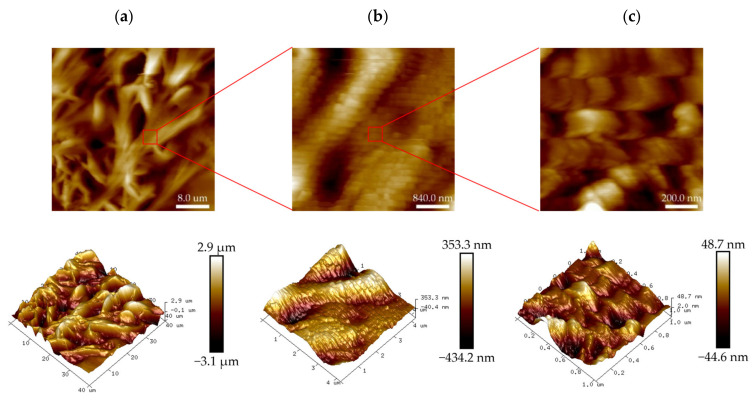
Two-dimensional (2D) and three dimensional (3D) images of the PHRO gel in deionized water with AFM. (**a**) Scanning area is 40.0 μm × 40.0 μm; (**b**) scanning area is 4.2 μm × 4.2 μm; (**c**) scanning area is 1.0 μm × 1.0 μm.

The microscopic morphology of the PHRO gel in deionized water is shown in Figure 3. The gel has a robust three-dimensional network structure like the “stem-leaf” of plants, and the network is embedded with granular substances of different sizes. It is speculated that the particle substance is phenolic resin with different molecular weights generated by the reaction of formaldehyde and RO. The particles could reinforce the strength of the gel structure. 

### 2.2. Effect of Additive Concentration on Gelling Performance of PHRO Gel

The gelation time, gel strength and dehydration rate of PHRO gels with different concentrations of PAM, HMTA, RO and OA were measured. The experimental temperature was 30 °C, and deionized water was used to prepare the liquid.

#### 2.2.1. Effect of PAM Concentration on Gelling Performance

**Figure 4:**
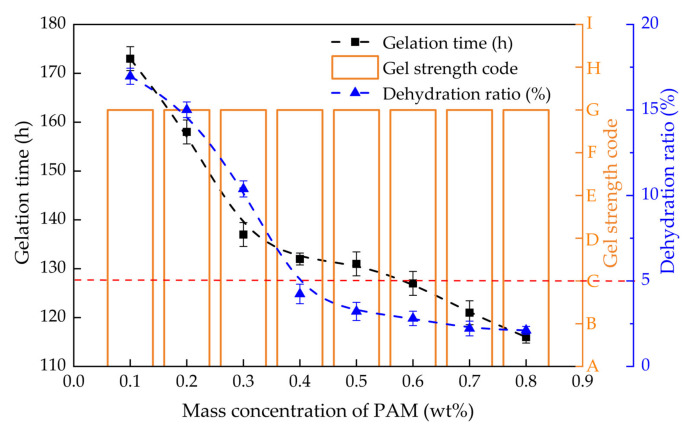
Gelling performance with different concentrations of PAM (0.5 wt% HMTA + 0.05 wt% RO + 0.3 wt% OA).

The gelation time, gel strength and dehydration rate of PHRO gels with different concentrations of PAM (0.1–0.8 wt%) were measured while keeping concentrations of HMTA, RO and OA constant, and the results are exhibited in Figure 4. With the increase in the concentration of PAM from 0.1 wt% to 0.8 wt%, the gel strength of PHRO remained at the “G” level. The gelation time was shortened from 173 h to 116 h, reduced by 32.9%. The dehydration rate declined by 14.9%, from 17.0% to 2.1%.

The results show that the increase in the concentration of PAM is beneficial for increasing the gelatinization rate and stability. This is because the number of “-CONH_2_” groups involved in crosslinking increases with increasing PAM concentration, resulting in a faster reaction rate and stronger crosslinking density. With the dehydration rate < 5.0% as the standard, the optimal PAM dosage is ≥ 0.4 wt%.

#### 2.2.2. Effect of HMTA Concentration on Gelling Performance

**Figure 5:**
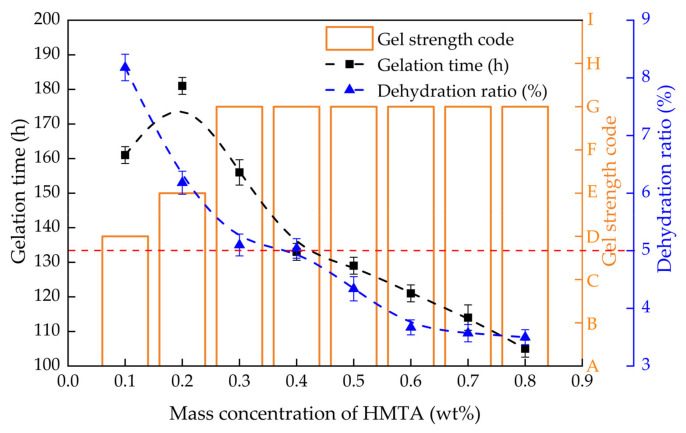
Gelling performance with different concentrations of HMTA (0.4 wt% PAM + 0.05 wt% RO + 0.3 wt% OA).

The gelation time, gel strength and dehydration rate of PHRO gels with different concentrations of HMTA (0.1–0.8 wt%) were measured while keeping concentrations of PAM, RO and OA constant, and the results are shown in Figure 5. When the concentration of HMTA was ≤0.2 wt%, the gel strength increased from “D” to “E”, the gelation time increased from 161 h to 181 h, and the dehydration rate decreased from 8.2% to 6.2%. When the concentration of HMTA was >0.2 wt%, the gel strength increased from “E” to “G”, the gelation time decreased from 181 h to 105 h, and the dehydration rate decreased from 6.2% to 3.5%.

The results show that a low concentration of HMTA could not prepare high-strength gels, and the increase in the concentration of HMTA is beneficial for increasing the gelatinization rate and stability. This is because when the concentration of HMTA is low, the number of “-OH” groups involved in crosslinking is low. The crosslinking density is poor, and the gel strength is weak. With the increase in HMTA concentration, the number of “-OH” groups increased, the crosslinking reaction accelerated, and the crosslinking density increased. With the dehydration rate < 5.0% as the standard, the optimal HMTA dosage is ≥ 0.5 wt%.

#### 2.2.3. Effect of RO Concentration on Gelling Performance

**Figure 6:**
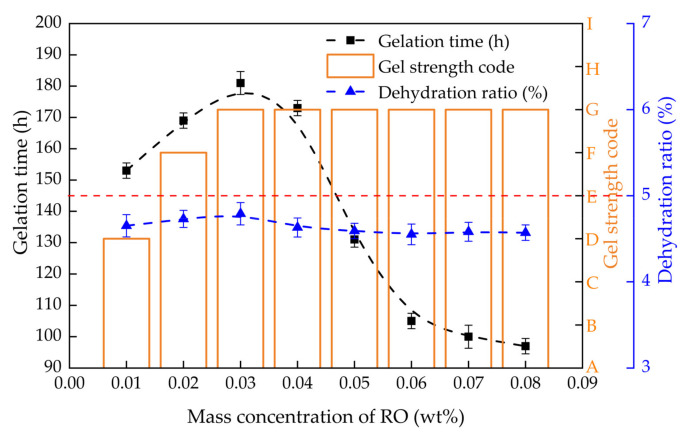
Gelling performance with different concentrations of RO (0.4 wt% PAM + 0.5 wt% HMTA + 0.3 wt% OA).

The gelation time, gel strength and dehydration rate of PHRO gels with different concentrations of RO (0.01–0.08 wt%) were measured while keeping concentrations of PAM, HMTA and OA constant, and the results are illustrated in Figure 6. When the RO concentration was ≤0.03 wt%, the gel strength increased from “D” to “G”. The gelation time increased from 153 h to 181 h, and the dehydration rate increased from 4.7% to 4.8%. When the RO concentration was >0.03 wt%, the gel strength remained at the “G” level. The gelation time was shortened from 181 h to 97 h, and the dehydration rate was reduced from 4.8% to 4.6%.

The results show that a low concentration of RO could not prepare high-strength gels. And increasing the concentration of RO is beneficial for increasing the gelatinization rate and stability. This is because with the increase in the concentration of RO, the contents of polyhydroxymethyl phenol and phenolic resin increased; that is, the number of crosslinking sites increased. Therefore, a low concentration of RO has low crosslinking density and weak gel strength, while a high RO concentration has a high crosslinking rate, high crosslinking density and high gel strength. The gelation time should be as short as possible in field applications, so the optimal RO dosage is ≥0.05 wt%.

#### 2.2.4. Effect of OA Concentration on Gelling Performance

**Figure 7:**
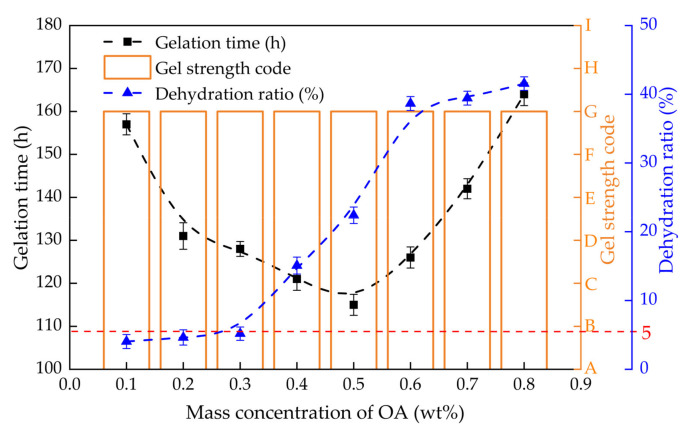
Gelling performance with different concentrations of OA (0.4 wt% PAM + 0.5 wt% HMTA + 0.05 wt% RO).

The gelation time, gel strength and dehydration rate of PHRO gels with different OA concentrations (0.1–0.8 wt%) were measured while keeping PAM, HMTA and RO concentrations constant, and the results are displayed in Figure 7. When the concentration of OA was ≤0.5 wt%, the gel strength remained at the “G” level, the gelation time was shortened from 157 h to 115 h, and the dehydration rate increased from 4.0% to 22.4%. When the concentration of was OA >0.5 wt%, the gel strength remained unchanged at the “G” level, the gelatinization time was extended from 115 h to 164 h, and the dehydration rate increased from 22.4% to 41.6%.

The results show that a low concentration of OA is beneficial for increasing the gelatinization rate and stability of gels, but a high concentration of OA is unfavorable. This is because the acidity of the solution increases with increasing OA concentration, which can accelerate the hydrolysis reaction of HMTA and thus accelerate the crosslinking reaction. When the concentration of OA is too high, the acid will destroy the structure of PAM, resulting in a slow crosslinking rate and a decrease in the stability of gels. With the dehydration rate < 5.0% as the standard, the optimal OA dosage is ≤0.3 wt%.

### 2.3. The Effect of Aqueous Salinity on Gelling Performance of PHRO Gel

The influence of Na^+^, K^+^, Ca^2+^ and Mg^2+^ on PHRO gelling performance was studied by measuring the gel strength, gelation time and dehydration rate of PHRO in different concentrations of NaCl, KCl, CaCl_2_ and MgCl_2_ solutions. The gel formula in the experiment was 0.4 wt% PAM + 0.5 wt% HMTA + 0.05 wt% RO + 0.3 wt% OA, and the experimental temperature was 30 °C.

#### 2.3.1. Gelling Performance in Monovalent Salt Solution

**Figure 8:**
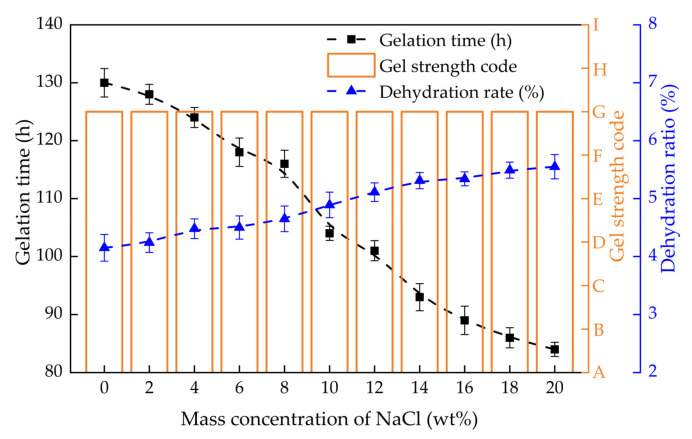
Gelling performance of PHRO in different concentrations of NaCl solution.

**Figure 9:**
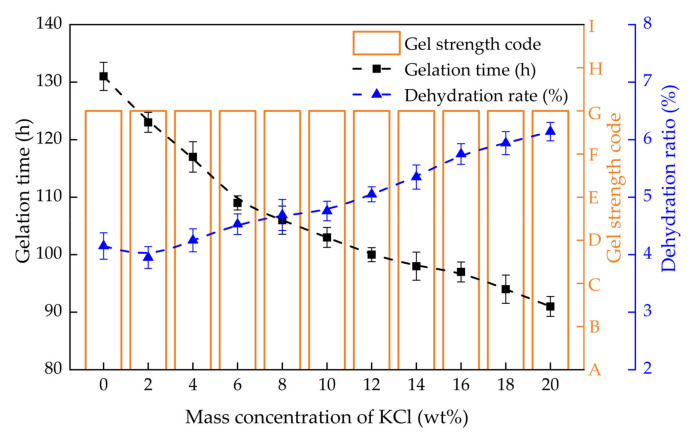
Gelling performance of PHRO in different concentrations of KCl solution.

The gel strength, gelation time and dehydration rate of PHRO gels in NaCl and KCl solutions with different concentrations (0–20.0 wt%) are shown in Figure 8 and Figure 9, respectively. With the increase in the concentration of NaCl, the gel strength remained at the “G” level, and the gelation time was reduced from 130 h to 84 h. The dehydration rate increased from 4.1% to 5.5%. With the increase in the concentration of KCl, the gel strength was maintained at the “G” level. The gelation time was reduced from 131 h to 91 h, and the dehydration rate increased from 4.1% to 6.1%.

The results indicate that PHRO can form high-strength gels and good stability in NaCl and KCl solutions with high concentrations; that is, the PHRO gels have good resistance to univalent salt ions. This is due to the electrostatic shielding effect of Na^+^ and K^+^, which can make the molecular chain of PAM curl up to a certain extent and shorten the distance between molecules [26]. However, this effect is slight and only accelerates the crosslinking reaction but has little negative influence on gel strength.

#### 2.3.2. Gelling Performance in Divalent Salt Solutions

**Figure 10:**
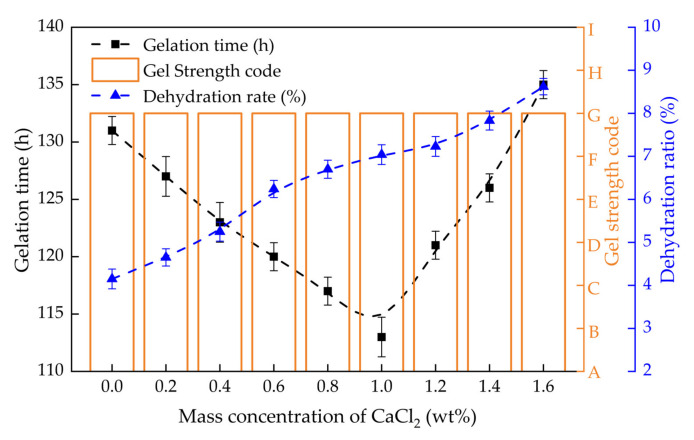
Gelling performance of PHRO in different concentrations of CaCl_2_ solution.

**Figure 11:**
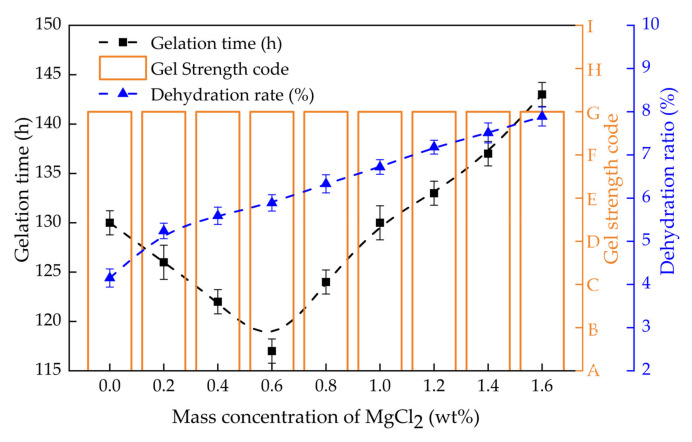
Dehydration rate of PHRO in different concentrations of MgCl_2_ solution.

The gel strength, gelation time and dehydration rate of PHRO gels in CaCl_2_ and MgCl_2_ solutions with different concentrations (0–1.6 wt%) are shown in Figure 10 and Figure 11, respectively. With the increase in the concentration of CaCl_2_, the gel strength remained at the “G” level, the gelation time was first shortened from 131 h to 115 h and then increased to 135 h, and the dehydration rate increased from 4.1% to 8.6%. Moreover, with the increase in the concentration of MgCl_2_, the gel strength remained at the “G” level, the gelation time was first shortened from 130 h to 117 h and then increased to 143 h, and the dehydration rate increased from 4.1% to 7.9%.

The results show that PHRO can form high-strength gels with good stability in high concentrations of CaCl_2_ and MgCl_2_ solutions; that is, PHRO gels have good resistance to bivalent saline ions. Ca^2+^ and Mg^2+^ have a stronger electrostatic shielding effect than Na^+^ and K^+^ [27,28]. When the concentration of CaCl_2_ and MgCl_2_ is low, they react with OA to form precipitation. As the concentration of Ca^2+^ and Mg^2+^ increases, the shielding effect of Ca^2+^ and Mg^2+^ on the PAM molecular chain is increased, which intensifies the curl of the PAM molecule. This tends to lead to internal crosslinking between “-CONH_2_” and “-COO^-^” on the PAM molecular chain, resulting in weakened inter-molecular crosslinking, and then the strength and stability of the gel are reduced.

### 2.4. Plugging Performance

**Figure 12:**
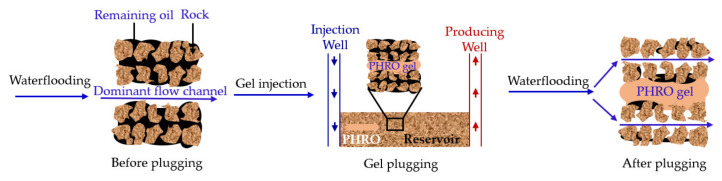
Schematic diagram of functional performance of PHRO gel in formation.

**Figure 13:**
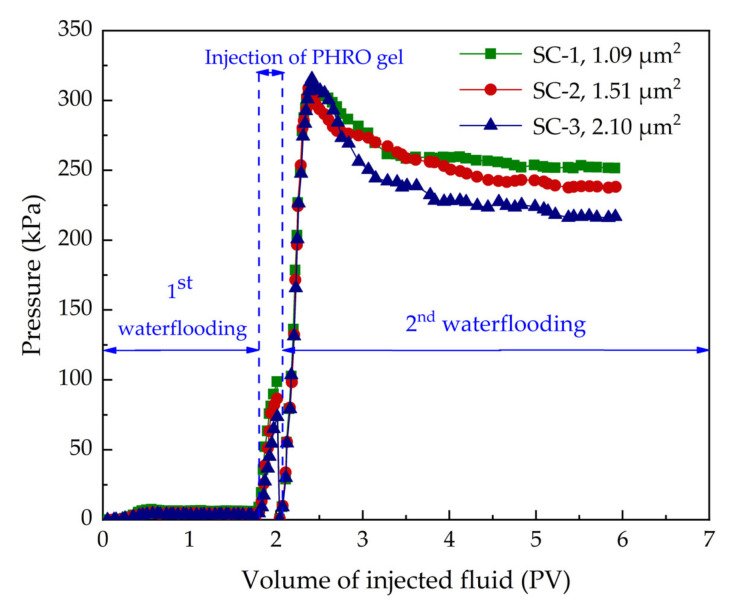
Pressure response of PHRO during conformance control experiment (the formula of gel is 0.4 wt% PAM + 0.5 wt% HMTA + 0.05 wt% RO + 0.3 wt% OA).

The functional performance of PHRO gel in underground formation is shown in Figure 12. The main process is as follows: (1) select the well group with abundant remaining oil; (2) according to the construction design scheme, inject PHRO uncrosslinked gel solution into the injection well; and (3) waterflooding again. The experimental results of the plugging performance of PHRO gel are shown in Figure 13 and Table 1. In the process of injection, the injection pressure increased rapidly from 73.6–98.7 kPa due to the high viscosity of the uncrosslinked gel solution, which increased by 70.2–92.6 kPa compared with that of the simulated formation water. After PHRO was gelatinized completely, the initial pressure increased to the highest point in the subsequent waterflooding process and then decreased slightly. As the pressure increased, the simulated formation water broke through part of the core channel, and the pressure stabilized when the simulated formation water was injected. The calculated residual resistance coefficient is higher than 41.0, and the plugging rate of the gel is higher than 97.6%. Moreover, the residual resistance coefficient and plugging rate increased with the increase in core permeability.

The results show that PHRO gel has good plugging performance and is suitable for conformance control in low-temperature and high-salinity formation.

### 2.5. Discussion

The effect of saline ions on PHRO gels is mainly on the PAM molecular chain. After PAM hydrolysis, the molecular chain contains negative “-COO^-^”, and the molecular chain is extended due to electrostatic repulsion [26]. According to the “Lyotropic series” theory and osmotic pressure theory, the electrostatic shielding of Na^+^, K^+^, Ca^2+^ and Mg^2+^ can weaken the intermolecular and intramolecular electrostatic repulsion of PAM [29]. As a result, PAM molecules get closer to each other, and their chains curl up. The strength of electrostatic shielding is related to the valence state and concentration of saline ions. To explore the salt tolerance mechanism of PHRO gel, the microtopography of PHRO in NaCl and CaCl_2_ solutions was observed.

**Figure 14:**
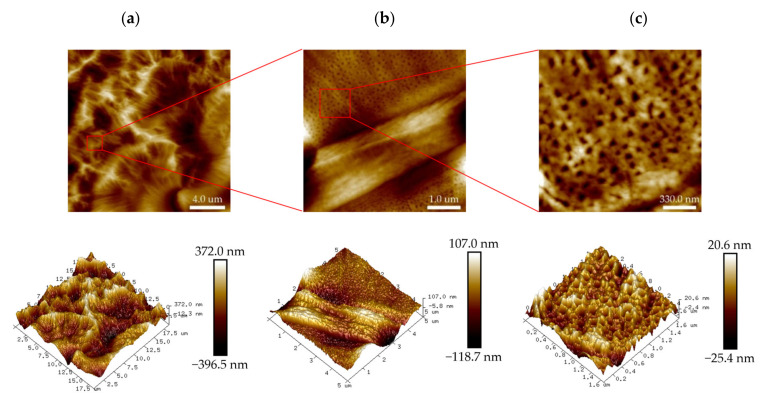
Two-dimensional (2D) and three-dimensional (3D) images of the PHRO gel in 20.0 wt% NaCl solution with AFM. (**a**) Scanning area is 18.8 μm × 18.8 μm; (**b**) scanning area is 5.0 μm × 5.0 μm; (**c**) scanning area is 1.7 μm × 1.7 μm.

Firstly, the microscopic morphology of PHRO in NaCl solution with a concentration of 20.0 wt% was investigated, and the results are shown in Figure 14. The PHRO gel has a complete network structure, and the mesh shrinks to form a denser network. The results show that the double-layer structure of PAM was not damaged in high-concentration sodium chloride solution, and there was still strong hydrogen bonding between PAM and water molecules. Therefore, the gel still has good stability.

**Figure 15:**
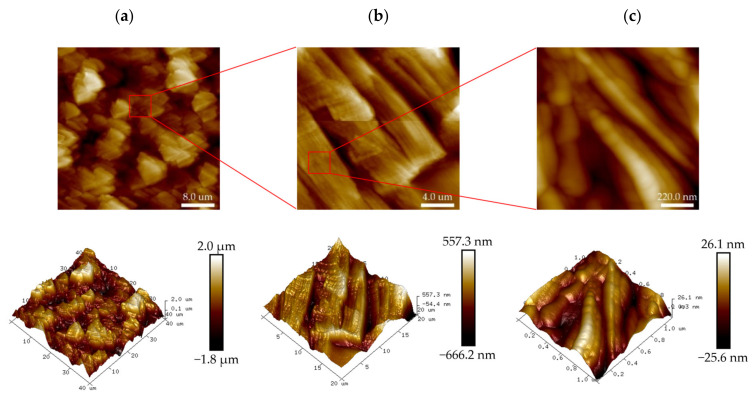
Two-dimensional (2D) and three-dimensional (3D) images of the PHRO gel in 1.6 wt% CaCl_2_ solution with AFM. (**a**) Scanning area is 40.0 μm × 40.0 μm; (**b**) scanning area is 20.0 μm × 20.0 μm; (**c**) scanning area is 1.1 μm × 1.1 μm.

Secondly, the microscopic morphology of PHRO in CaCl_2_ solution with a concentration of 1.6 wt% was scanned, as shown in Figure 15. The PHRO gel has a complete network structure, with more mesh shrinkage and lower mesh numbers than those in NaCl solution. This is because the electrostatic shielding effect of Ca^2+^ is much stronger than that of Na^+^, the molecular chain of PAM is more curled up, and the hydrogen bonding between PAM and water molecules is more damaged. However, due to the shielding effect of OA on Ca^2+^, the gel still has good stability in saline water.

There are two reasons that PHRO gel has good salt resistance. On the one hand, formaldehyde and resorcinol react to produce phenolic resin particles of different molecular weights, which are evenly dispersed in the network structure of PHRO. These particles cover the PAM skeleton and attain a saline ion shielding effect. On the other hand, the addition of OA can partially eliminate the influence of Ca^2+^ and Mg^2+^ and improve the stability of gels.

In conclusion, the PHRO gel has good salt resistance, and appropriate salinity can accelerate the crosslinking reaction and improve the stability of gels. The results show that it is suitable for conformance control in high-salinity formation. In addition, gels of different strengths can be prepared by adjusting the concentration of the polymer and crosslinking agent. During the field application, the high-strength gel blocks the near-well zone and the weak gel blocks the in-depth zone to realize in-depth profile control.

## 3. Conclusions

A PHRO gel suitable for conformance control of low-temperature and high-salinity reservoirs was prepared, and the influence of the concentration of each component on the gelling performance of the gel was investigated. The gelling performance of PHRO in sodium chloride, potassium chloride, calcium chloride and magnesium chloride solutions were studied. The microstructure of PHRO in sodium chloride and calcium chloride solution was observed, and the salt resistance mechanism of PHRO gel was analyzed. Some conclusions may be drawn as follows:

(1) PHRO can form gels with high strength and good stability at a low temperature. The optimal concentrations of different additives were PAM ≥ 0.4 wt%, HMTA≥ 0.5 wt%, RO ≥ 0.05 wt% and OA ≤ 0.3 wt%. The strength of the gel can reach the “G” grade. The gelation time is 105–173 h, and the dehydration rate is 2.1–5.2%.
(2) The strength of PHRO gels in different concentrations of NaCl and KCl solutions can reach the “G” level, and the dehydration rate is 4.1–6.1%. The gelatinization time is 84–131 h, and it decreases with the increase in saline ion concentration. Furthermore, the strength of PHRO gels in different concentrations of CaCl_2_ and MgCl_2_ solutions can be maintained at the “G” level, and the dehydration rate is 4.1–8.6%. The gelatinization time is 115–143 h, and it decreases with the increase in saline ion concentration.
(3) PHRO gel has a robust “stem-leaf” three-dimensional network structure, which contains embedded phenolic resin with different sizes and different molecular weights. It can enhance the strength of the PAM skeleton and improve the stability of the gel. Moreover, the PHRO gel still has a complete network structure in a high-concentration salt solution, the network is more compact due to mesh contraction, and the gel structure still has high strength.
(4) The plugging rate of PHRO in simulated formation water is more than 97.6%, which is suitable for conformance control in low-temperature and high-salinity reservoirs.
(5) The salt-resistant performance of PHRO gel is mainly because the phenolic resin generated by the fluid can strengthen the gel network structure, and OA has the function of shielding salt ions. The appropriate number of saline ions is beneficial for accelerating the crosslinking reaction and improving the gel strength. The results show that PHRO gel is suitable for in-depth conformance control in low-temperature and high-salinity formation, which has broad application prospects.

## 4. Materials and Methods

### 4.1. Materials

#### 4.1.1. Additives

The polymer used in this study was PAM with an average molecular weight of 1.0 × 10^7^ g/mol and a hydrolysis degree of 3.6%, which was manufactured by Anhui Tianrun Chemical Industry Co., Ltd. (Bengbu, China). The crosslinking agents used were HMTA and RO, which were provided by Weifang Xingjia Chemical Co., Ltd. (Weifang, China). To encourage the fluid to crosslink at low temperatures, OA was used as a cross-promoting agent, purchased from Shandong Feishuo Chemical Technology Co., Ltd. (Jinan, China). The salts used for the salt resistance study fluid were NaCl, KCl, CaCl_2_, NaHCO_3_ and MgCl_2_·6H_2_O, which were obtained from Pharmaceutical Group Chemical Reagents Co., Ltd. (Shanghai, China). The water used in the experiment was deionized water.

#### 4.1.2. Simulated Formation Water

The simulated formation water used in the experiment in this paper was prepared according to the ionic composition of formation water from North Buzachi Oilfield. The total salinity of the simulated water was 63,246.2 mg/L, and the ionic composition is shown in Table 2.

### 4.2. Methods

#### 4.2.1. Preparation of PHRO Gel

The PHRO gel was prepared at room temperature (25 °C). First, PAM was evenly dispersed in the solution according to the designed dosage and stirred with a mechanical stirrer (IKA RW20, IKA, Königswinter, Germany) at a rate of 300 r/min for 2 h. Then, HMTA, RO and OA were successively added according to the designed dosage and stirred at a rate of 300 r/min for 30 min. Finally, 15 mL of the well-stirred initial solution of the gel was put into an ampoule bottle, sealed with an alcohol burner and placed in the oven (UFP 500, Memmert Company, Schwabach, Germany) at 30 °C.

#### 4.2.2. Determination of Gelation Time and Gel Strength

In this study, the GSC strength code method was used to determine the strength of PHRO [30,31]. Letters A-I were used to represent different strength levels of the gel, and grade “I” was the highest strength, as shown in Table 3. First, the initial solution of PHRO gel was prepared according to the method in Section 4.2.1. Secondly, the samples were taken out of the oven every hour to record the time and strength code. When the strength code no longer changes, the current code is the strength level of PHRO, and the time is the gelation time of PHRO. There were three parallel samples in each group, and the gelation time was the average value of the sample data.

#### 4.2.3. Stability of PHRO Gel

In this study, the dehydration rate ($D_{w}$) was used to characterize the stability of PHRO gel. The lower the $D_{w}$ of PHRO gel in the same aging time, the better its stability. First, the initial solution of PHRO gel was prepared according to the method in Section 4.2.1., and the mass of gel-forming solution was recorded as $m_{0}$. Secondly, the prepared samples were aged for 90 days in the oven at 30 °C. Finally, the mass of the solid phase in the ampoule bottle aged for 90 days was weighed at room temperature (25 °C) and denoted as $m_{1}$. There were three parallel samples in each group, and the dehydration rate was the average value of the sample data. The calculation formula of $D_{w}$ is:

(1) $$\begin{array}{c} D_{w}=\frac{m_{0}-m_{1}}{m_{0}}\times 100\% \end{array}$$

#### 4.2.4. Plugging Performance of PHRO Gel

The plugging performance of PHRO gels is characterized by the plugging rate (E) and residual resistance coefficient ($F_{RR}$). The higher the E and $F_{RR}$, the better the plugging performance.

**Figure 16:**
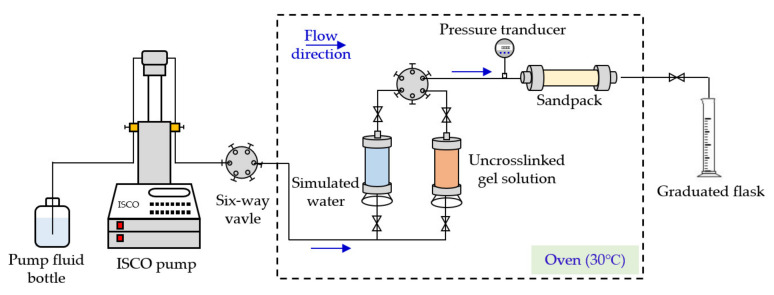
Flow chart of sandpack model.

The plugging performance of PHRO gels was evaluated by using a sandpack with a diameter of 25 mm and a length of 200 mm. The flow chart of the evaluation device is shown in Figure 16. Sandpacks with different permeabilities were obtained by filling them with quartz sand with different sizes, and 300 mesh nets for sand leaking prevention were placed at the inlet and outlet of the sandpack to prevent sand production. The porosity and permeability parameters of the sandpack are shown in Table 4. The plugging evaluation experiments of gels were carried out in the oven at 30 °C, and the fluid injection rate was 1.0 mL/min.

First, the simulated formation water was injected, and the stable pressure $P_{1}$ was recorded after the pressure stabilized. Secondly, the uncrosslinked gel solution of PHRO gel was prepared according to the method in Section 4.2.1. Thirdly, 0.3 PV (pore volume) of the initial solution of the gel was injected, and the sandpack was placed in the oven and aged for 7 days. Finally, simulated formation water was injected, and the stable pressure $P_{2}$ was recorded after the pressure stabilized.

The calculation formula of the residual resistance factor is:

(2) $$\begin{array}{c} F_{RR}=\frac{P_{2}}{P_{1}} \end{array}$$

The calculation formula of the plugging rate is:

(3) $$\begin{array}{c} E=\left(1-\frac{P_{1}}{P_{2}}\right)\times 100\% \end{array}$$

#### 4.2.5. The Microscopic Morphology of PHRO Gel

Atomic force microscopy (AFM) (Multimode 8, Bruker Company, Billerica, MA, USA) was used to observe the microstructure of PHRO gels. First, the initial solution of PHRO gel was prepared as described in Section 4.2.1. Secondly, an appropriate amount of gel sample was cut and placed on a copper sheet with scissors to make it spread into a film as thin as possible and dried in a freeze dryer (SCIENTZ-10N, Ningbo Scientz Biotechnology Co., Ltd., Ningbo, China) for 24 h. Finally, the microstructure of PHRO was observed in air in AFM tapping mode, and the related parameters were determined. The experimental temperature was 30 °C, and the room humidity was 30%.

## Figures and Tables

**Table 1:** Plugging ability of gels under different permeability conditions.

Number of Sandpack	$P_{1}\;(\mathrm{kPa})$	$P_{2}\;(\mathrm{kPa})$	$F_{RR}$	*E* (%)
SC-1	6.13	251.6	41.0	97.6
SC-2	4.23	238.2	56.3	98. 2
SC-3	3.31	216.8	62.5	98.5

**Table 2:** Ionic composition of simulated formation water.

Ionic Type	Cl^−^	SO_4_^2−^	HCO_3_^−^	Mg^2+^	Ca^2+^	Na^+^	K^+^
Ionic content (mg/L)	36879.4	540.9	72.6	579.3	1985	17445.9	5743.1

**Table 3:** Gel strength code [30,31].

Gel Strength Code	Gel Category	Gel Description
A	No detectable gel	The same viscosity as the original polymer solution.
B	Highly flowing gel	Only slightly more viscous than the initial polymer solution.
C	Flowing gel	Most of the gel flows to the bottle cap by gravity upon inversion.
D	Moderately flowing gel	Only a small portion (5.0–10.0%) of the gel does not readily flow to the bottle cap by gravity upon inversion
E	Barely flowing gel	Significant portion (>15.0%) of the gel does not flow by gravity upon inversion.
F	Highly deformable non-flowing gel	The gel does not flow to the bottle cap by gravity upon inversion.
G	Moderately deformable non-flowing gel	The gel deforms about halfway down the bottle by gravity upon inversion.
H	Slightly deformable non-flowing gel	Only the gel surface slightly deforms by gravity upon inversion.
I	Rigid gel	There is no gel surface deformation by gravity upon inversion.

**Table 4:** Porosity and permeability of sandpack.

Number of Sandpack	Permeability (μm^2^)	Porosity (%)
SC-1	1.09	25.4
SC-2	1.51	29.3
SC-3	2.10	31.0

## Data Availability

Data are contained within the article.
